# Refractory to Improvement: A Cautionary Tale of Two-Electron Densities Computed with One-Electron Basis Sets and of Their Employment in Quantum-Chemical Formalisms

**DOI:** 10.1021/acs.jctc.6c00413

**Published:** 2026-07-23

**Authors:** Jerzy Cioslowski, Javier Domin, Eduard Matito

**Affiliations:** † Institute of Physics, 49673 University of Szczecin, Wielkopolska 15, Szczecin 70-451, Poland; ‡ Max-Planck-Institut für Physik Komplexer Systeme, Nöthnitzer Straße 38, Dresden 01187, Germany; § 226245 Donostia International Physics Center (DIPC), Donostia, Euskadi 20018, Spain; ∥ Donostia Polimero eta Material Aurreratuak: Fisika, Kimika eta Teknologia, Kimika Fakultatea, Euskal Herriko Unibertsitatea EHU, Donostia, Euskadi 20080, Spain; ⊥ Ikerbasque Foundation for Science, Bilbao, Euskadi 48009, Spain

## Abstract

A report on detailed investigations of the properties of the approximate on-top two-electron densities Φ­(*r⃗*) computed with one-electron basis functions is presented. The key findings of these investigations, which also concern the reduced variant of Φ­(*r⃗*), i.e., $\phi \left(\mathrm{r⃗}\right)=\frac{4\Phi \left(\mathrm{r⃗}\right)}{{\left[\rho_{1}(\mathrm{r⃗})\right]}^{2}}$ [where ρ_1_(*r⃗*) is the one-electron density], are as follows: 1) Φ­(*r⃗*) converge extremely slowly to the complete basis set (CBS) limits, their errors asymptotically scaling $∼N^{-1/3}$ with the number N of basis functions (as contrasted with the $∼N^{-1}$ error scaling of the electronic energy); 2) this slow convergence results in gross inaccuracies of the approximate Φ­(*r⃗*) computed with standard basis sets; 3) the errors in these Φ­(*r⃗*) are, in general, refractory to reduction with standard convergence acceleration techniques such as the CBS-limit extrapolation; 4) it is essential to make a distinction between the physically meaningful genuine Φ­(*r⃗*) (such as those produced by highly accurate calculations) and the contrived ones (such as those appearing in some variants of the Kohn–Sham formalism and in overly simplistic models) that are merely auxiliary quantities to which no physical meaning should be attached; 5) due to the presence of spurious features in the contrived Φ­(*r⃗*), their employment in the analysis of electronic structure leads to false and misleading conclusions on chemical bonding and electron correlation; and 6) the MC-PDFT formalism suffers from a hidden “double-counting” problem, whose presence is masked for sufficiently small active spaces by its use of the contrived Φ­(*r⃗*).

## Graphical abstract

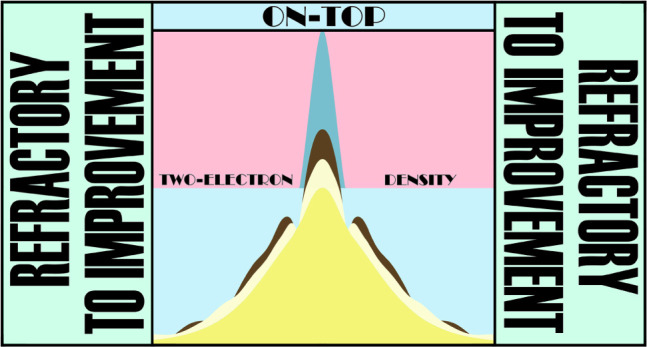

## 1 Introduction

Accurate numerical evaluation of various quantities related to the two-electron density is a prerequisite for the proper treatment of electron correlation and thus credible prediction of electronic properties. However, despite their pivotal role in both computational and interpretive aspects of electronic structure theory, surprisingly little attention has been paid to the critical assessment of the errors imparted to these quantities by various approximations employed in actual quantum-chemical calculations. This lack of attention has produced a fertile ground for the construction of oversimplistic models yielding *qualitatively* incorrect two-electron densities, whose spurious features have, in turn, led to false conclusions concerning both chemical bonding and electron correlation.

The emergence of numerous published studies affected by these flaws has prompted the investigations reported in this paper. A detailed overview of the interrelations among the various quantities of importance to these investigations is presented below in order to facilitate the understanding of the resultant theoretical arguments and numerical data.

Since all the measurable properties of assemblies of Coulombically interacting particles are given by expectation values of one- and two-body operators, any stationary state of an atom, a molecule, or an extended system is wholly described within the nonrelativistic Born–Oppenheimer approximation by the pertinent two-electron reduced density matrix (the 2-matrix)^1,2^

1 Γ2≡Γ2(x1,x2;x1′,x2′)=N(N−1)2∫...∫Ψ*(x1′,x2′,x3,...,xN)×Ψ(x1,x2,x3,...,xN)dx3...dxN

derived from the underlying single-component *N*-electron wave function Ψ ≡ Ψ­(***x***_1_, ***x***_2_, ***x***_3_, ..., ***x***_*N*_) that is an eigenfunction of the spin operators *Ŝ*_*z*_ and *Ŝ* ^2^. However, as only the one-electron reduced density matrix (the 1-matrix)

2 Γ1≡Γ1(x1;x1′)=2N−1∫Γ2(x1,x2;x1′,x2)dx2=N∫...∫Ψ*(x1′,x2,x3,...,xN)×Ψ(x1,x2,x3,...,xN)dx2...dxN

and the diagonal part of ^2^Γ, i.e., the two-electron (a.k.a. pair) density

3 ρ2≡ρ2(x1,x2)=Γ2(x1,x2;x1,x2)=N(N−1)2∫...∫Ψ*(x1,x2,x3,...,xN)×Ψ(x1,x2,x3,...,xN)dx3...dxN

enter the expressions for the expectation values of, respectively, an arbitrary one-electron operator and the electron-electron repulsion energy *W*, the knowledge of the entire 2-matrix is usually not necessary except for the seldom instances involving two-electron properties other than *W*. Moreover, it is, in general, preferable to employ the cumulant of the two-electron density (note its sign convention that is commonly used in quantum chemistry but is opposite to that used by physicists)^3,4^

4 $$D_{2}\equiv D_{2}\left(x_{1},x_{2}\right)=\rho_{2}\left(x_{1},x_{2}\right)-\frac{1}{2}\;\rho_{1}\left(x_{1}\right)\;\rho_{1}\left(x_{2}\right)$$

where

5 ρ1≡ρ1(x1)=Γ1(x1;x1)=N∫...∫Ψ*(x1,x2,x3,...,xN)×Ψ(x1,x2,x3,...,xN)dx2...dxN

is the diagonal part of ^1^Γ, i.e., the one-electron density, instead of ρ_2_. This preference stems from the additive separabilities

6 Γ1[AB](x1;x1′)=Γ1[A](x1;x1′)+Γ1[B](x1;x1′)

and

7 $$D_{2[AB]}\left(x_{1},x_{2}\right)=D_{2[A]}\left(x_{1},x_{2}\right)+D_{2[B]}\left(x_{1},x_{2}\right)$$

where the subscripted letters in square brackets refer to either two noninteracting systems *A* and *B* or the respective combined system *AB*, of ^1^Γ and D . It follows from eqs 2–5 that ^1^Γ and D are not fully independent as

8 Γ1(x1;x1)=ρ1(x1)=−2∫D2(x1,x2)dx2

Both Γ1(x1;x1′) and $D_{2}\left(x_{1},x_{2}\right)$ are given by sums involving products of Cartesian and spin components, i.e.

Γ1(x1;x1′)=Γ1αα(r⃗1;r⃗1′) α(σ1) α*(σ1′)+Γ1ββ(r⃗1;r⃗1′) β(σ1) β*(σ1′) 9

and

10 $$D_{2}\left(x_{1},x_{2}\right)=D_{2}^{\alpha \alpha }\left({\mathrm{r⃗}}_{1},{\mathrm{r⃗}}_{2}\right)\;\left|\alpha \left(\sigma_{1}\right){|}^{2}\;\right|\alpha \left(\sigma_{2}\right){|}^{2}+D_{2}^{\beta \beta }\left({\mathrm{r⃗}}_{1},{\mathrm{r⃗}}_{2}\right)\;\left|\beta \left(\sigma_{1}\right){|}^{2}\;\right|\beta \left(\sigma_{2}\right){|}^{2}+D_{2}^{\alpha \beta }\left({\mathrm{r⃗}}_{1},{\mathrm{r⃗}}_{2}\right)\;\left|\alpha \left(\sigma_{1}\right){|}^{2}\;\right|\beta \left(\sigma_{2}\right){|}^{2}+D_{2}^{\beta \alpha }\left({\mathrm{r⃗}}_{1},{\mathrm{r⃗}}_{2}\right)\;\left|\beta \left(\sigma_{1}\right){|}^{2}\;\right|\alpha \left(\sigma_{2}\right){|}^{2}+D_{2}^{ms}\left({\mathrm{r⃗}}_{1},{\mathrm{r⃗}}_{2}\right)\;\alpha \left(\sigma_{1}\right)\;\beta^{*}\left(\sigma_{1}\right)\;\beta \left(\sigma_{2}\right)\;\alpha^{*}\left(\sigma_{2}\right)+D_{2}^{ms}\left({\mathrm{r⃗}}_{2},{\mathrm{r⃗}}_{1}\right)\;\beta \left(\sigma_{1}\right)\;\alpha^{*}\left(\sigma_{1}\right)\;\alpha \left(\sigma_{2}\right)\;\beta^{*}\left(\sigma_{2}\right)$$

from which their spin-summed variants

11 Γ1(r⃗1;r⃗1′)=Γ1αα(r⃗1;r⃗1′)+Γ1ββ(r⃗1;r⃗1′)

and

12 $$D_{2}\left({\mathrm{r⃗}}_{1},{\mathrm{r⃗}}_{2}\right)=D_{2}^{\alpha \alpha }\left({\mathrm{r⃗}}_{1},{\mathrm{r⃗}}_{2}\right)+D_{2}^{\beta \beta }\left({\mathrm{r⃗}}_{1},{\mathrm{r⃗}}_{2}\right)+D_{2}^{\alpha \beta }\left({\mathrm{r⃗}}_{1},{\mathrm{r⃗}}_{2}\right)+D_{2}^{\beta \alpha }\left({\mathrm{r⃗}}_{1},{\mathrm{r⃗}}_{2}\right)$$

arise [note the different arguments employed and the absence of the cumulant $D_{2}^{ms}\equiv D_{2}^{ms}\left({\mathrm{r⃗}}_{1},{\mathrm{r⃗}}_{2}\right)$ , which originates from the αβαβ spin block of the 2-matrix and thus does not survive the spin summation]. The spin resolution of ^1^Γ translates into that of ρ_1_, namely,

13 $$\rho_{1}\left(x_{1}\right)=\rho_{1}^{\alpha }\left({\mathrm{r⃗}}_{1}\right)\;\left|\alpha \left(\sigma_{1}\right){|}^{2}+\rho_{1}^{\beta }\left({\mathrm{r⃗}}_{1}\right)\;\right|\beta \left(\sigma_{1}\right){|}^{2}$$

giving rise to the definition $\rho_{1}\left({\mathrm{r⃗}}_{1}\right)=\rho_{1}^{\alpha }\left({\mathrm{r⃗}}_{1}\right)+\rho_{1}^{\beta }\left({\mathrm{r⃗}}_{1}\right)$ . This spin-summed one-electron density is normalized to *N*, whereas for the spin density $\rho_{1}^{s}\left({\mathrm{r⃗}}_{1}\right)=\rho_{1}^{\alpha }\left({\mathrm{r⃗}}_{1}\right)-\rho_{1}^{\beta }\left(\overset{\to }{r_{1}}\right)$ one has

14 $$\int \rho_{1}^{s}\left({\mathrm{r⃗}}_{1}\right)\;d{\mathrm{r⃗}}_{1}=2\;\left\langle \Psi \right|{Ŝ}_{z}\left|\Psi \right\rangle$$

The additive separability of the linear combination

15 $$\rho_{1}^{lc}\left({\mathrm{r⃗}}_{1}\right)=\lambda^{\alpha }\rho_{1}^{\alpha }\left({\mathrm{r⃗}}_{1}\right)+\lambda^{\beta }\rho_{1}^{\beta }\left({\mathrm{r⃗}}_{1}\right)$$

with arbitrary real-valued coefficients λ^α^ and λ^β^ follows from eqs 5, 6, and 9. Thanks to the zero differential overlap (ZDO), i.e., the vanishing of the product $\rho_{1[A]}^{lc}\left({\mathrm{r⃗}}_{1}\right)\;\rho_{1[B]}^{lc}\left({\mathrm{r⃗}}_{1}\right)$ for all *r⃗*_1_, $\rho_{1}^{lc}\left({\mathrm{r⃗}}_{1}\right)$ is also spatially separable. Since the ZDO property also holds for a product of two such linear combinations with different sets of coefficients, any polynomial in $\rho_{1}^{\alpha }\left({\mathrm{r⃗}}_{1}\right)$ and $\rho_{1}^{\beta }\left({\mathrm{r⃗}}_{1}\right)$ [such as, e.g., $\rho_{1}\left({\mathrm{r⃗}}_{1}\right)$ , $\rho_{1}^{s}\left({\mathrm{r⃗}}_{1}\right)$ , and $\rho_{1}^{\alpha }\left({\mathrm{r⃗}}_{1}\right)\;\rho_{1}^{\beta }\left({\mathrm{r⃗}}_{1}\right)$ ] is both additively and spatially separable.

Being six-dimensional objects, neither the individual Cartesian components of Γ1(x1;x1′) and $D_{2}\left(x_{1},x_{2}\right)$ nor their spin-summed variants are readily amenable to straightforward analysis or visualization. However, the spectral decompositions of ^1^Γ (in terms of the natural orbitals and their occupation numbers introduced over 70 years ago^5^) and $D_{2}$ (in terms of the natural densitals and their amplitudes proposed only very recently^6^) produce quantities well suited for both computational and interpretive quantum-chemical formalisms.

The intracule density^7,8^

16 $$I\equiv I\left(\mathrm{r⃗}\right)=\int \int \rho_{2}\left({\mathrm{r⃗}}_{1},{\mathrm{r⃗}}_{2}\right)\;\delta \left({\mathrm{r⃗}}_{1}-{\mathrm{r⃗}}_{2}-\mathrm{r⃗}\right)\;d{\mathrm{r⃗}}_{1}\;d{\mathrm{r⃗}}_{2}$$

where δ­(*r⃗*) is the three-dimensional Dirac delta, its spherical average

17 $$\mathrm{I̅}\equiv \mathrm{I̅}\left(r\right)=\frac{1}{4\pi }\int_{0}^{2\pi }\left[\int_{0}^{\pi }I\left(r\mathrm{e⃗}\right)\;\sin\theta \;d\theta \right]\;d\varphi$$

where e⃗=(sinθcosφ,sinθsinφ,cosθ) , and the on-top two-electron (a.k.a. pair) density

18 $$\Phi \equiv \Phi \left(\mathrm{r⃗}\right)=\rho_{2}\left(\mathrm{r⃗},\mathrm{r⃗}\right)=\rho_{1}^{\alpha }\left(\mathrm{r⃗}\right)\;\rho_{1}^{\beta }\left(\mathrm{r⃗}\right)+D_{2}^{↑↓}\left(\mathrm{r⃗},\mathrm{r⃗}\right)$$

where

19 $$D_{2}^{↑↓}\equiv D_{2}^{↑↓}\left({\mathrm{r⃗}}_{1},{\mathrm{r⃗}}_{2}\right)=D_{2}^{\alpha \beta }\left({\mathrm{r⃗}}_{1},{\mathrm{r⃗}}_{2}\right)+D_{2}^{\beta \alpha }\left({\mathrm{r⃗}}_{1},{\mathrm{r⃗}}_{2}\right)$$

derive from the spin-summed two-electron (a.k.a. pair) density

20 $$\rho_{2}\left({\mathrm{r⃗}}_{1},{\mathrm{r⃗}}_{2}\right)=\frac{1}{2}\;\rho_{1}\left({\mathrm{r⃗}}_{1}\right)\;\rho_{1}\left({\mathrm{r⃗}}_{2}\right)+D_{2}\left({\mathrm{r⃗}}_{1},{\mathrm{r⃗}}_{2}\right)$$

The integral identities

21 $$\int I\left(\mathrm{r⃗}\right)\;d\mathrm{r⃗}=4\pi \int \mathrm{I̅}\left(r\right)\;r^{2}\;dr=\frac{1}{2}\;N\left(N-1\right)$$

and

22 $$\int \frac{I\left(\mathrm{r⃗}\right)}{\left|\mathrm{r⃗}\right|}\;d\mathrm{r⃗}=4\pi \int \mathrm{I̅}\left(r\right)\;r\;dr=W$$

follow directly from eq 1. In addition, I̅ conforms to both the cusp condition^9^

23 $$\frac{d\mathrm{I̅}\left(r\right)}{dr}{|}_{\;r=0}=\mathrm{I̅}\left(0\right)$$

which reflects the behavior of both ρ_2_(*r⃗*_1_,*r⃗*_2_) and $D_{2}\left({\mathrm{r⃗}}_{1},{\mathrm{r⃗}}_{2}\right)$ at *r⃗*_2_ → *r⃗*_1_, and to the normalization of Φ

24 ∫Φ(r⃗)dr⃗=I̅(0)

Being genuine densities, I(r⃗) , I̅(r⃗) , and Φ­(*r⃗*) are nonnegative-valued at all *r⃗*. The additive and spatial separabilities of Φ stem from those of its components [i.e., the product $\rho_{1}^{\alpha }\left(\mathrm{r⃗}\right)\;\rho_{1}^{\beta }\left(\mathrm{r⃗}\right)$ and the cumulant $D_{2}^{↑↓}\left(\mathrm{r⃗},\mathrm{r⃗}\right)$ ] that enter the r.h.s. of eq 14. On the other hand, although both I(r⃗) and I̅(r⃗) are additively separable thanks to the presence of the three-dimensional Dirac delta in the integral that appears in the r.h.s. of eq 12, neither of them is spatially separable. Consequently, unlike in the cases of ρ_1_ and Φ, the patterns of critical points and gradient lines in the intracule densities $I_{\left[A\right]}$ and $I_{\left[B\right]}$ of two noninteracting subsystems *A* and *B* are not discernible in their counterpart $I_{\left[AB\right]}$ pertaining to the respective combined system *AB*. For this reason, despite their interesting (and aesthetically pleasing) morphologies,^10–12^ these patterns are suitable as interpretive tools only for species involving a few electrons.

The cumulant $D_{2}^{↑↓}$ that enters eqs 14 and 15 has the spectral decomposition

25 $$D_{2}^{↑↓}\left({\mathrm{r⃗}}_{1},{\mathrm{r⃗}}_{2}\right)=\underset{n}{\sum }f_{n}^{↑↓}\;d_{n}^{↑↓}\left({\mathrm{r⃗}}_{1}\right)\;d_{n}^{↑↓}\left({\mathrm{r⃗}}_{2}\right)$$

in terms of the respective natural densitals $\left\{d_{n}^{↑↓}\right\}\equiv \left\{d_{n}^{↑↓}\left(\mathrm{r⃗}\right)\right\}$ and their amplitudes $\left\{f_{n}^{↑↓}\right\}$ .^6^ The condition

26 $$\int d_{n}^{↑↓}\left(\mathrm{r⃗}\right)\;d\mathrm{r⃗}=0$$

is satisfied for all *n*, implying the presence of at least one nodal surface in every natural densital. Combining eqs 14 and 21 produces

27 $$\Phi \left(\mathrm{r⃗}\right)=\rho_{1}^{\alpha }\left(\mathrm{r⃗}\right)\;\rho_{1}^{\beta }\left(\mathrm{r⃗}\right)+\underset{n}{\sum }f_{n}^{↑↓}\;{\left[d_{n}^{↑↓}(\mathrm{r⃗})\right]}^{2}$$

The relative spin polarization η ≡ η­(*r⃗*) and the reduced on-top two-electron density ϕ ≡ ϕ­(*r⃗*) are defined by the ratios

28 $$\eta \left(\mathrm{r⃗}\right)=\frac{\rho_{1}^{s}\left(\mathrm{r⃗}\right)}{\rho_{1}\left(\mathrm{r⃗}\right)}$$

and

29 $$\phi \left(\mathrm{r⃗}\right)=\frac{4\Phi \left(\mathrm{r⃗}\right)}{{\left[\rho_{1}(\mathrm{r⃗})\right]}^{2}}$$

respectively. Both η and ϕ are additively and spatially separable. Combining eqs 14 and 23–25 yields

$$\phi \left(\mathrm{r⃗}\right)=1-{\left[\eta (\mathrm{r⃗})\right]}^{2}+\frac{4D_{2}^{↑↓}\left(\mathrm{r⃗},\mathrm{r⃗}\right)}{{\left[\rho_{1}(\mathrm{r⃗})\right]}^{2}}=1-{\left[\eta (\mathrm{r⃗})\right]}^{2}+4\underset{n}{\sum }f_{n}^{↑↓}\;{[\frac{d_{n}^{↑↓}\left(\mathrm{r⃗}\right)}{\rho_{1}\left(\mathrm{r⃗}\right)}]}^{2}$$ 30

In the case of the underlying Ψ being a single determinant, $D_{2}^{↑↓}\left(\mathrm{r⃗},\mathrm{r⃗}\right)$ is identically equal to zero and thus ϕ­(*r⃗*) is fully determined by η­(*r⃗*), being identically equal to one in the absence of spin polarization.

All of the aforediscussed quantities are functionals of Ψ. In the following, this dependence is explicitly indicated by the notation ρ_1_[Ψ] ≡ ρ_1_[Ψ]­(*r⃗*), etc., whenever deemed beneficial for clarity.

The stage is now set for the investigation of how various concepts and formalisms of the electronic structure theory are affected by the insufficient accuracies of the aforedefined quantities due to the inadequate approximations employed in their computation.

## 2 The Diverse Facets of the On-Top Two-Electron Density

The on-top two-electron density (and its reduced variant) is encountered in quantum-chemical formalisms far more often than the other incarnations of ρ_2_ mentioned in the Introduction. The context in which Φ appears in these formalisms determines the varying requirements for its accuracy that ensure the absence of artifacts in the computed electronic properties.

Entering the off-diagonal cusp condition for ^1^Γ,^13,14^ Φ entirely controls the leading asymptotics of the natural orbitals and their occupation numbers at the limit of the latter tending to zero,^15^ which permits its reconstruction from these quantities^16^ (note that the natural densitals and their amplitudes exhibit analogous asymptotics in terms of Φ^6^). The broad consequences of this unexpected connection range from a generalization of the Hill asymptotics^17^ to a universal power law governing the accuracy of wave function-based electronic structure calculations^18^ and symmetry equiincidence of natural orbitals.^19^ In these formalisms, replacing the exact on-top two-electron density by its approximate counterpart has a negligible effect of practical importance due to the fact that the relevant multiplicative constants involving integrals of Φ are either replaced by fitted values anyway [as in the cases in which they provide justification for the complete basis set (CBS) extrapolation schemes^17,18^ ] or cancel out altogether (as in the case of the symmetry equiincidence principle^19^).

The on-top two-electron density also appears in a reformulation of the Kohn–Sham (KS) variant^20^ of the density functional theory (DFT) whose simplest implementation is based upon the local spin-density approximation (LSDA), in which the exchange-correlation component *E*_*xc*_ ≡ *E*_*xc*_[ρ_1_, η] of the electronic energy is given by^21^

31 $$E_{xc}\left[\rho_{1},\eta \right]\approx {Ẽ}_{xc}^{LSDA}\left[\rho_{1},\eta \right]=\int \rho_{1}\left(\mathrm{r⃗}\right)\;\epsilon_{xc}^{HEG}\left(\rho_{1}\left(\mathrm{r⃗}\right),\eta \left(\mathrm{r⃗}\right)\right)\;d\mathrm{r⃗}$$

where $\epsilon_{xc}^{HEG}\left(\rho_{1},\eta \right)$ is the exchange-correlation energy per particle of a homogeneous electron gas (HEG) with the (position-independent) ρ_1_ and η. As the negative-valued $\epsilon_{xc}^{HEG}\left(\rho_{1},\eta \right)$ is a monotonically decreasing function of η^2^,^22,23^ global minima of approximate electronic energies computed with the exchange-correlation component given by ${Ẽ}_{xc}^{LSDA}\left[\rho_{1},\eta \right]$ do not always correspond to η­(*r⃗*) being identically equal to zero in spin-unpolarized systems. The existence of such minima poses the “symmetry dilemma” of choosing between the spin-unpolarized solution of the KS equations that yields grossly inaccurate electronic energy and the broken-symmetry one that often affords surprisingly small energy error at the cost of unphysical η­(*r⃗*).^24^ The triplet instabilities that lead to this dilemma are typically encountered in “abnormal” systems^25^ (e.g., the archetypal H_2_ molecule with a stretched bond, as well as the C_2_ and Cr_2_ molecules at equilibrium geometries), in which, unlike in their “normal” (e.g., the He atom) and “mildly abnormal” (e.g., the Be atom) counterparts, nondynamical electron correlation is prominent. An “escape” from the symmetry dilemma is offered by an elegant conceptual maneuver,^25,26^ in which η is relegated to the role of an auxiliary quality that, instead of producing physically relevant spin density $\rho_{1}^{s}\left(\mathrm{r⃗}\right)=\eta \left(\mathrm{r⃗}\right)\rho_{1}\left(\mathrm{r⃗}\right)$ , yields the reduced on-top two-electron density within the LSDA approximation

32 $$\phi \left(\mathrm{r⃗}\right)\approx {\mathrm{\phi ̃}}^{LSDA}\left(\mathrm{r⃗}\right)=\left(1-{\left[\eta (\mathrm{r⃗})\right]}^{2}\right)\;g^{HEG}\left(\rho_{1}\left(\mathrm{r⃗}\right),\eta \left(\mathrm{r⃗}\right);0\right)$$

where $g^{HEG}\left(\rho_{1},\eta ;r\right)$ is the pair-correlation function of HEG with the (position-independent) ρ_1_ and η.^22^ Although systematic studies of the accuracy of the on-top two-electron densities that follow from such approximate ϕ have not been carried out thus far, these quantities have been found to reproduce certain integrals of Φ quite faithfully (at least for the “normal” systems).^18,25,26^ Since in this case Φ is the product of the reformulated KS formalism rather than an input for it, its accuracy (or its lack) does not affect at all those of the other electronic properties (i.e., the electronic energy and the one-electron density) computed in this manner.

The employment of the fictitious relative spin polarization as the proxy for nondynamical correlation does not prevent other types of artifacts, such as symmetry mismatches between the one-electron density and the external potential due to nuclei, from occurring in KS calculations involving both the LSDA^26^ and more sophisticated functionals.^27^ However, it has potential advantages that go beyond the circumvention of the triplet instability problem described above. In particular, it provides the means for restoring the proper energy ordering of states with different multiplicities that is incorrectly predicted within the original KS formalism. This point is vividly illustrated by an example involving several approximate two-electron wave functions

33 $$\Psi_{I}\left(x_{1},x_{2}\right)=\frac{1}{\sqrt{2}}\;\varphi_{1}\left({\mathrm{r⃗}}_{1}\right)\;\varphi_{1}\left({\mathrm{r⃗}}_{2}\right)\times \left[\alpha \left(\sigma_{1}\right)\;\beta \left(\sigma_{2}\right)-\beta \left(\sigma_{1}\right)\;\alpha \left(\sigma_{2}\right)\right]$$

$$\Psi_{II}\left(x_{1},x_{2}\right)=\frac{1}{2}\;\left[\varphi_{1}\left({\mathrm{r⃗}}_{1}\right)\;\varphi_{2}\left({\mathrm{r⃗}}_{2}\right)+\varphi_{2}\left({\mathrm{r⃗}}_{1}\right)\;\varphi_{1}\left({\mathrm{r⃗}}_{2}\right)\right]\times \left[\alpha \left(\sigma_{1}\right)\;\beta \left(\sigma_{2}\right)-\beta \left(\sigma_{1}\right)\;\alpha \left(\sigma_{2}\right)\right]$$ 34

35 $$\Psi_{III}\left(x_{1},x_{2}\right)=\frac{1}{\sqrt{2}}\;\left[\varphi_{1}\left({\mathrm{r⃗}}_{1}\right)\;\varphi_{2}\left({\mathrm{r⃗}}_{2}\right)-\varphi_{2}\left({\mathrm{r⃗}}_{1}\right)\;\varphi_{1}\left({\mathrm{r⃗}}_{2}\right)\right]\times \alpha \left(\sigma_{1}\right)\;\alpha \left(\sigma_{2}\right)$$

36 $$\Psi_{IV}\left(x_{1},x_{2}\right)=\frac{1}{2}\;\left[\varphi_{1}\left({\mathrm{r⃗}}_{1}\right)\;\varphi_{2}\left({\mathrm{r⃗}}_{2}\right)-\varphi_{2}\left({\mathrm{r⃗}}_{1}\right)\;\varphi_{1}\left({\mathrm{r⃗}}_{2}\right)\right]\times \left[\alpha \left(\sigma_{1}\right)\;\beta \left(\sigma_{2}\right)+\beta \left(\sigma_{1}\right)\;\alpha \left(\sigma_{2}\right)\right]$$

and

37 $$\Psi_{V}\left(x_{1},x_{2}\right)=\frac{1}{\sqrt{2}}\;\left[\varphi_{1}\left({\mathrm{r⃗}}_{1}\right)\;\varphi_{2}\left({\mathrm{r⃗}}_{2}\right)-\varphi_{2}\left({\mathrm{r⃗}}_{1}\right)\;\varphi_{1}\left({\mathrm{r⃗}}_{2}\right)\right]\times \beta \left(\sigma_{1}\right)\;\beta \left(\sigma_{2}\right)$$

listed in Table 1. The first of these wave functions, which describes a singlet state arising from two electrons occupying the same orbital φ_1_ ≡ φ_1_(*r⃗*), yields vanishing η­(*r⃗*) and $D_{2}^{↑↓}\left(\mathrm{r⃗},\mathrm{r⃗}\right)$ , which in turn implies ϕ­(*r⃗*) = 1 at all *r⃗*. The other four wave functions, which stem from two electrons occupying two different orbitals φ_1_ ≡ φ_1_(*r⃗*) and φ_2_ ≡ φ_2_(*r⃗*), give rise to identical one-electron densities, thus forming within the KS formalism two distinct doublets with η­(*r⃗*) = 0 and η­(*r⃗*) = ±1, respectively. The first of these doublets comprises two double-determinant wave functions, namely the open-shell singlet Ψ_*II*_ ≡ Ψ_*II*_(***x***_1_, ***x***_2_) and the triplet Ψ_*IV*_ ≡ Ψ_*IV*_(***x***_1_, ***x***_2_), whereas the second one is composed of two single-determinant wave functions Ψ_*III*_ ≡ Ψ_*III*_(***x***_1_, ***x***_2_) and Ψ_*V*_ ≡ Ψ_*V*_(***x***_1_, ***x***_2_) pertaining to the triplet state. Needless to say, the wave functions Ψ_*II*_ and Ψ_*IV*_ involve nondynamical electron correlation, whereas those of the doublet Ψ_*III*_/Ψ_*V*_ are completely uncorrelated. Accordingly, in contrast to those pertaining to Ψ_*III*_ and Ψ_*V*_, the cumulants $D_{2}^{↑↓}\left(\mathrm{r⃗},\mathrm{r⃗}\right)$ corresponding to Ψ_*II*_ and Ψ_*IV*_ assume, in general, nonzero values (always nonpositive for the latter and possibly positive for the former).

**Table 1:** One- and Two-Electron Densities Corresponding to Various Approximate Two-Electron Wave Functions

$\Psi \left(x_{1},x_{2}\right)$	$\rho_{1}\left(\mathrm{r⃗}\right)$	η(r⃗)	$D_{2}^{↑↓}\left(\mathrm{r⃗},\mathrm{r⃗}\right)$	ϕ(r⃗)	$\eta^{•}\left(\mathrm{r⃗}\right)$
Ψ_*I*_(***x***_1_, ***x***_2_)	2 |φ_1_(*r⃗*)|^2^	0	0	1	0
Ψ_*II*_(***x***_1_, ***x***_2_)[Table-fn tbl1fn2]	|φ_1_(*r⃗*)|^2^ + |φ_2_(*r⃗*)|^2^	0	$\frac{1-2f\left(\mathrm{r⃗}\right)}{4}\;{\left[\rho_{1}(\mathrm{r⃗})\right]}^{2}$	2 – 2 *f*(*r⃗*)	$\pm \sqrt{2f\left(\mathrm{r⃗}\right)-1}$
Ψ_*III*_(***x***_1_, ***x***_2_)	|φ_1_(*r⃗*)|^2^ + |φ_2_(*r⃗*)|^2^	1	0	0	±1
Ψ_*IV*_(***x***_1_, ***x***_2_)	|φ_1_(*r⃗*)|^2^ + |φ_2_(*r⃗*)|^2^	0	$-\frac{1}{4}\;{\left[\rho_{1}(\mathrm{r⃗})\right]}^{2}$	0	±1
Ψ_*V*_(***x***_1_, ***x***_2_)	|φ_1_(*r⃗*)|^2^ + |φ_2_(*r⃗*)|^2^	–1	0	0	±1

a $f\left(\mathrm{r⃗}\right)={\left[\frac{{\left|\varphi_{1}(\mathrm{r⃗})\right|}^{2}-{\left|\varphi_{2}\left(\mathrm{r⃗}\right)\right|}^{2}}{{\left|\varphi_{1}\left(\mathrm{r⃗}\right)\right|}^{2}+{\left|\varphi_{2}(\mathrm{r⃗})\right|}^{2}}\right]}^{2}$ .

The spurious energy ordering that emerges within the conventional KS formalism, namely the degeneracy of the open-shell singlet with one of the triplets and the splitting of the triplet spin sublevels, is lifted upon replacing η­(*r⃗*) with^28^

38 $$\eta^{•}\equiv \eta^{•}\left(\mathrm{r⃗}\right)=\pm \sqrt{1-\phi \left(\mathrm{r⃗}\right)}=\pm \sqrt{{\left[\eta (\mathrm{r⃗})\right]}^{2}-4\;\frac{D_{2}^{↑↓}\left(\mathrm{r⃗},\mathrm{r⃗}\right)}{{\left[\rho_{1}(\mathrm{r⃗})\right]}^{2}}}$$

In the case of uncorrelated wave functions, η and η^•^ coincide as $D_{2}^{↑↓}\left(\mathrm{r⃗},\mathrm{r⃗}\right)$ is identically equal to zero. Otherwise, |η^•^(*r⃗*)| > η­(*r⃗*) when $D_{2}^{↑↓}\left(\mathrm{r⃗},\mathrm{r⃗}\right)<0$ (which is a typical situation encountered for e.g., Ψ_*IV*_ in the above example) and |η^•^(*r⃗*)| < η­(*r⃗*) when $D_{2}^{↑↓}\left(\mathrm{r⃗},\mathrm{r⃗}\right)$ is both positive-valued and not greater than $\frac{1}{4}\;{\left[\eta (\mathrm{r⃗})\;\rho_{1}(\mathrm{r⃗})\right]}^{2}$ . In the case of $D_{2}^{↑↓}\left(\mathrm{r⃗},\mathrm{r⃗}\right)>\frac{1}{4}\;{\left[\eta (\mathrm{r⃗})\;\rho_{1}(\mathrm{r⃗})\right]}^{2}$ , η^•^(*r⃗*) becomes imaginary-valued, which does not pose numerical problems as all the known approximate functionals for *E*_*xc*_ are even functions of η.

Since the three triplet wave functions Ψ_*III*_, Ψ_*IV*_, and Ψ_*V*_ produce identical |*η* ^•^| that differs from that corresponding to the open-shell singlet Ψ_*II*_, replacing η with η^•^ restores the proper energy ordering. Therefore, one may be inclined to regard this replacement as a definite improvement that, via a fictitious relative spin polarization, makes it possible to account for the nondynamical correlations present in “abnormal” systems while not affecting their “normal” counterparts. However, a closer look at this approach reveals several caveats of crucial importance. First of all, it is unclear how to handle the imaginary-valued η^•^ that, although yielding real-valued *E*_*xc*_[ρ_1_, η^•^] (within both LSDA and more sophisticated approximations), cannot be ascribed any obvious physical meaning. Even worse, since the employment of η^•^ hinges upon the availability of the on-top two-electron density from an external quantum-chemical formalism, the accuracy of the resultant electronic properties is bound to depend strongly on that of the input Φ.

As illustrated by an example involving the wave function Ψ_*I*_ listed in Table 1, this dependence has deep conceptual roots and thus is more than a purely numerical issue. Being completely uncorrelated, Ψ_*I*_ yields a vanishing cumulant $D_{2}^{↑↓}\left(\mathrm{r⃗},\mathrm{r⃗}\right)$ , and thus the reduced on-top two-electron density ϕ­(*r⃗*) that is identically equal to one. In other words, Ψ_*I*_ describes a “normal” system with vanishing true and fictitious relative spin polarizations. Therefore, for two-electron states with reasonable zeroth-order descriptions provided by Ψ_*I*_, KS calculations involving *E_xc_* [*ρ*_1_, *η* ^•^] are expected to afford rather accurate predictions for the one-electron densities and the electronic energies. Unfortunately, since Ψ_*I*_ is devoid of the cusp at *r⃗*_2_ → *r⃗*_1_, this fidelity of description does not carry over to ϕ that arises from the two-electron density evaluated at the electron-electron coalescence point. Thus, the constant ϕ­(*r⃗*) equal to one is completely fake even for the ground state of the helium atom (which is an archetypal “normal” system), as the actual ϕ­(*r⃗*) assumes values not exceeding ca. 0.6 (Figure 1). Consequently, one is faced with the dilemma of using either the contrived ϕ set to one that yields a reasonable electronic energy or the true ϕ that, by giving rise to the fictitious relative spin polarization characteristic of the “abnormal” systems, is bound to produce a vastly exaggerated correlation energy. Whereas the latter choice is unacceptable on practical grounds, the arbitrariness and inherent inconsistency of the former one makes it equally unpalatable. The inconsistency in question is readily revealed upon the inspection of *ϕ̃* ^*LSDA*^(*r⃗*) [with *g* ^*HEG*^(ρ_1_, η; 0) that enters eq 28 obtained from a published interpolation formula^22^] afforded by KS calculations employing the contrived ϕ. Although differing significantly from the true ϕ­(*r⃗*), *ϕ̃* ^*LSDA*^(*r⃗*) is still significantly smaller than one at all *r⃗* (Figure 1). Interestingly, it yields the estimate (see eq 20) of I̅(0)≈0.12043804 that, when juxtaposed with its essentially exact counterpart of I̅(0)≈0.10634537 , turns out to be more accurate than e.g., I̅(0)≈0.12056335 computed within the full configuration interaction (FCI) formalism used in conjunction with the cc-pV5Z basis set (see the subsequent section of this paper).

**Figure 1:**
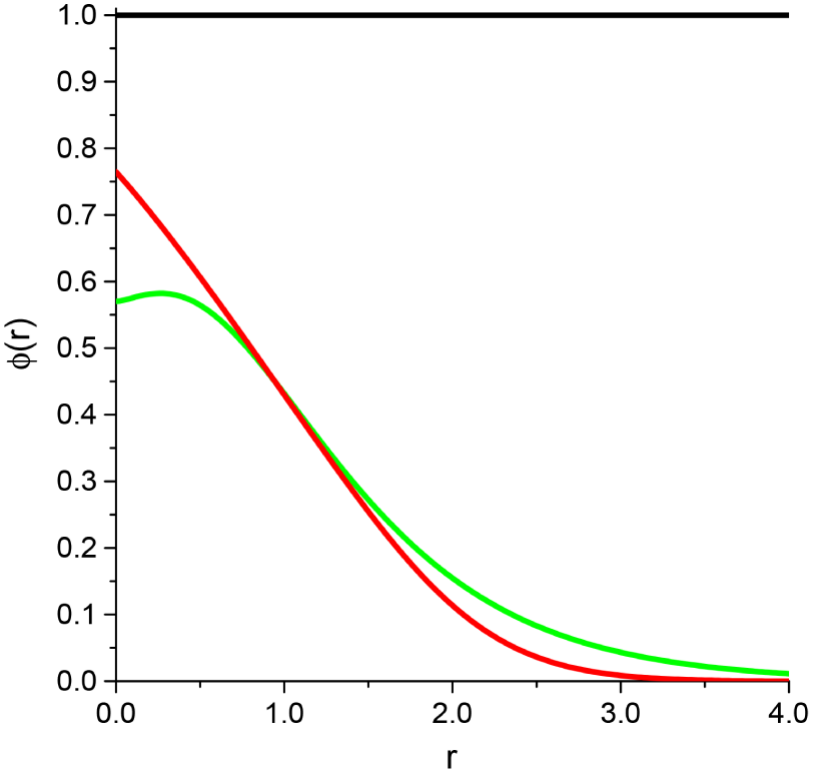
The reduced on-top two-electron density ϕ­(*r*) ≡ ϕ­(*r⃗*) with *r* = |*r⃗*| of the ground state of the helium atom. Green: the essentially exact ϕ­(*r*), red: *ϕ̃^LSDA^* (*r*) (eq 28), black: the fake ϕ­(*r*) contrived from Ψ*_I_* (Table 1).

In summary, by invoking the fictitious relative spin polarization defined by eq 29, one crosses the line between escaping the symmetry dilemma and procuring the on-top two-electron densities from quantum-chemical approaches external to the KS formalism. When coming from oversimplistic models, these densities have little in common with their exact counterparts. Thus, by relying on such models, one falls into a trap of employing contrived on-top two-electron densities that are of neither physical relevance nor interpretive value despite yielding reasonable predictions of some electronic properties upon being input into KS calculations. Even worse, using their accurate counterparts in this role is bound to produce vastly exaggerated electron correlation energies of singlet states that are uniformly treated as “abnormal” in such a case (note that e.g., two-electron triplet states, whose Φ vanish due to the antisymmetry of the underlying wave function, are free of this problem). This refractoriness to improvement exhibited by the KS formalism is a harbinger of even more acute difficulties encountered in attempts to conflate the conventional wave function-based approaches with the DFT ones.

The electronic energies of atoms and molecules computed with finite sets of one-electron basis functions converge in a notoriously slow manner to their CBS limits, the energy errors per electron being proportional to the respective ratios $\frac{N}{N}$ of the number of electrons *N* and the basis set size N .^18^ Fortunately, as the relative rather than absolute energies are directly comparable to the experimental data, a great deal of error cancellation is enjoyed in calculations involving species with sufficiently similar characteristics of electron correlation. However, this assumption does not hold in a significant proportion of cases, necessitating the employment of methods specialized toward efficient handling of nondynamical correlation effects. Among these, the complete active space self-consistent field (CASSCF) approach is especially useful. In this approach, each of the available orbitals is assigned to one of two sets, namely the active and core/virtual spaces, to which the FCI and Hartree–Fock (HF) formalisms are applied, respectively. A simultaneous minimization of the electronic energy with respect to both the FCI coefficients and all the orbitals yields the CASSCF wave function Ψ^*CASSCF*^ for a given one-electron basis set and a particular assignment of orbitals to the active space. This formalism has well-defined basis-set convergence properties, as assigning all of the available orbitals to the active space (i.e., attaining the FCI limit) produces the electronic energy that becomes exact at the CBS limit.

Although CASSCF calculations involving large active spaces are as prohibitively expensive as their FCI counterparts, nondynamical correlation effects can be accurately captured with small sets of active orbitals. However, as none or little dynamical correlation is accounted for in the resulting Ψ^*CASSCF*^, such an approach produces insufficiently accurate predictions of electronic properties. In order to alleviate this deficiency in a computationally inexpensive manner, efforts have been made toward devising schemes in which the CASSCF energies are augmented with contributions obtained within the KS formalism. Unfortunately, the progress in the construction of viable schemes of this type, which has been an active field of research since 1974,^29^ remains impeded by the “double-counting” problem that stems from the vagueness of the partitioning of the correlation energy into its dynamical and nondynamical components. As the number of orbitals assigned to the active space increases, Ψ^*CASSCF*^ evolves from that comprising almost exclusively (nearly-)­degenerate Slater determinants (e.g., those emerging at the bond dissociation limits) to that involving all the correlation that can be described within a particular one-electron basis set. Ideally, the KS energy contribution should concurrently be reduced from that encompassing all the dynamical correlation, which presumably is completely missing in the minimal-active-space Ψ^*CASSCF*^, to zero. Alas, this is not the case with any of the presently known conflations of the CASSCF and KS formalisms, some of which employ the on-top two-electron density as one of the central variables.^30^

One such approach is the MC-PDFT formalism, in which the electronic energy *E* ≡ *E*[Ψ] is approximated as^31^

$$E\approx E^{MC-PDFT}\equiv E^{MC-PDFT}\left[\Psi \right]=\left\langle \Psi \right|Ĥ\left|\Psi \right\rangle -\int \int \frac{D_{2}\left[\Psi \right]\left({\mathrm{r⃗}}_{1},{\mathrm{r⃗}}_{2}\right)}{\left|{\mathrm{r⃗}}_{1}-{\mathrm{r⃗}}_{2}\right|}\;d{\mathrm{r⃗}}_{1}\;d{\mathrm{r⃗}}_{2}+E_{ot}\left[\rho_{1}\left[\Psi \right],\eta^{•}\left[\Psi \right]\right]$$ 39

where Ψ is an approximate wave function of the CASSCF variety, *Ĥ* is the electronic Hamiltonian, and η^•^[Ψ] ≡ η^•^[Ψ]­(*r⃗*) is computed from η­[Ψ]­(*r⃗*), $D_{2}^{↑↓}\left[\Psi \right]\left(\mathrm{r⃗},\mathrm{r⃗}\right)$ , and ρ_1_[Ψ]­(*r⃗*) via eq 29. Various variants of the “on-top” functional *E*_*ot*_[ρ_1_,η^•^] are derived from their DFT counterparts via an *ad hoc* “translation” that involves the replacements $\rho_{1}^{s}\left(\mathrm{r⃗}\right)\to \eta^{•}\left(\mathrm{r⃗}\right)\;\rho_{1}^{s}\left(\mathrm{r⃗}\right)$ and $\mathrm{\nabla ⃗}\rho_{1}^{s}\left(\mathrm{r⃗}\right)\to \eta^{•}\left(\mathrm{r⃗}\right)\;\mathrm{\nabla ⃗}\rho_{1}^{s}\left(\mathrm{r⃗}\right)$ in their arguments. This “translation” is entirely empirical and, in fact, inconsistent, as $\eta^{•}\left(\mathrm{r⃗}\right)\;\mathrm{\nabla ⃗}\rho_{1}^{s}\left(\mathrm{r⃗}\right)$ does not equal $\mathrm{\nabla ⃗}\left[\eta^{•}\left(\mathrm{r⃗}\right)\;\rho_{1}^{s}\left(\mathrm{r⃗}\right)\right]$ (thus introducing a spurious dependence of *E*_*ot*_[ρ_1_, η^•^] on η). It should also be mentioned that a safeguard against imaginary-valued η^•^(*r⃗*) (i.e., using only its real part), employed in the original translations,^31^ has subsequently been found not only unnecessary but also precluding the proper description of certain electronic states.^32^

These nuances notwithstanding, by relying on the fictitious relative spin polarization, the approximation [Disp-formula eq30] inherits all the deficiencies from the aforediscussed modification of the KS formalism. These deficiencies are common to both the original (in which Ψ is the minimizer of ⟨Ψ|*Ĥ*|Ψ⟩)^31^ and the fully variational (in which Ψ is the minimizer of *E* ^*MC‑PDFT*^[Ψ])^33^ incarnations of the MC-PDFT approach. They are readily revealed by a careful analysis of the handling of the “normal” and “abnormal” systems within active spaces of different sizes. Consider first the case of a null active space, where the variational MC-PDFT formalism reverts to its conventional KS counterpart as η^•^ becomes identical with η. For “normal” systems, this implies quite accurate predictions of the electronic energies and the one-electron densities, whereas for the “abnormal” ones, the symmetry dilemma (with all its consequences, such as the spurious symmetry breaking and the incorrect multiplet splittings) reappears. Moving to small active spaces results in dramatic improvements for the latter, as the dependence of *E*_*ot*_[ρ_1_, η^•^] on the on-top two-electron density suppresses the instabilities responsible for the symmetry dilemma and enhances the exchange-correlation contribution to the electronic energy, purportedly compensating for the neglect of its nondynamical correlation component within the conventional approximate DFT functionals. On the other hand, this enhancement is an unwelcome development in the case of “normal” systems that are already described reasonably well by the “untranslated” functionals. As the size of the active space grows, so does the contribution of the dynamical correlation to the on-top two-electron density and thus to η^•^, amplifying this enhancement even further regardless of the presence/absence of nondynamical electron correlation. Consequently, at the FCI limit, the MC-PDFT formalism is expected to produce far less accurate electronic energies than its KS counterpart (certainly for the “normal” systems and most probably for the “abnormal” ones), thus being incapable of reproducing the exact energies at the CBS limit.

What transpires from the above analysis is that, quite peculiarly, the underlying concept of the MC-PDFT functionals boils down to an attempt at improving the slow convergence of the electronic energy with respect to the size of the active space by employing a property, namely the one-top two-electron density, that (as demonstrated in the subsequent section of this paper) converges even more slowly. Ironically, it is this exceedingly slow convergence of Φ that, by deferring the spurious overestimation of the electron correlation energy to larger active sets, rescues this scheme from a complete failure. Thus, it appears that the empirical success of the MC-PDFT calculations stems from the combination of a genuine improvement (at least for small active spaces) in the treatment of “abnormal” systems and a fortuitous cancellation of errors, which is a textbook example of a phenomenon known as the Pauling point.^34^ Simply put, despite claims to the contrary,^35^ the replacement of the CASSCF exchange-correlation contributions [described by the double integral involving $D_{2}$ that enters eq 30] with the “on-top” functional, merely conceals the double-counting problem that ultimately creeps back via the aforediscussed spurious enhancement. With this problem unsolved, one comes a full circle, arriving back at the original advice of using only small active spaces^29^ (which appears to be supported by some benchmark runs of MC-PDFT calculations^32,36^ ).

The above considerations are equally relevant to another class of approaches, such as the CASΠDFT,^37^ in which the CASSCF energies are corrected with add-on functionals (constructed from their conventional DFT counterparts) of ρ_1_ and Φ. In those formalisms, the amelioration of the double-counting problem requires a progressive attenuation of these add-on contributions upon the expansion of the active space, which necessitates piling additional *ad hoc* parameters dependent on the active-space size (and possibly also on the specific basis set used) upon those already plentifully present in the conventional functionals.^38^ Despite this evident overparameterization, the CASΠDFT method performs poorly in comparison with its MC-PDFT counterpart.^30^ An entirely different approach that relies upon the weak overlap of the spatial domains pertaining to the dynamical correlation (i.e., the relatively short-ranged Coulomb hole) and some instances of the nondynamical correlation (i.e., the long-range entanglement in the case of bond dissociation), should also be mentioned here. This range-separation partitioning, which comes with its own set of challenges,^30,39^ has been recently combined with the “on-top” exchange-correlation functionals.^40^ In summary, even when combined, the CASSCF and KS formalisms remain refractory to improvements of practical significance for the universal treatment of electron correlation.

The gross dissimilitudes between the on-top two-electron densities appearing in the aforediscussed approaches and those derived from sufficiently accurate wave functions make the former completely unsuitable for the description of correlation and bonding. Therefore, those densities have to be relegated to the role of auxiliary quantities (much like the fictitious relative spin polarization) that under no circumstances should be used in the construction of interpretive quantum-chemical tools, lest such tools lead to haphazard inferences concerning various aspects of electronic structure. Unfortunately, such inferences have been repeatedly made in the recent literature,^41–47^ presumably due to the general lack of familiarity with the properties of the two-electron density at the electron-electron coalescence points. In order to illustrate their pitfalls, it is instructive to examine the singlet ground states of the two simplest two-electron systems, namely, the He atom and the H_2_ molecule. This choice of systems permits the employment of the mutually complementary analytic instrumentation^15,48,49^ and very accurate numerical methods.^50,51^ It also offers the opportunity to elaborate upon the less detailed (though still convincing) results of a recent study.^52^

The electronic states in question are described by wave functions with the spectral representation^48^

40 $$\Psi \left(x_{1},x_{2}\right)=\underset{n}{\sum }\lambda_{n}\;\varphi_{n}\left({\mathrm{r⃗}}_{1}\right)\;\varphi_{n}\left({\mathrm{r⃗}}_{2}\right)\times \frac{1}{\sqrt{2}}\;\left[\alpha \left(\sigma_{1}\right)\;\beta \left(\sigma_{2}\right)-\beta \left(\sigma_{1}\right)\;\alpha \left(\sigma_{2}\right)\right]$$

where {φ_*n*_} ≡ {φ_*n*_(*r⃗*)} and {λ_*n*_} are, respectively, the natural orbitals (NOs) and the corresponding natural amplitudes (NAs), both real-valued and ordered nonascendingly in |λ_*n*_|. The spin-summed one-electron density and its on-top two-electron counterpart that follow from this wave function read

$$\rho_{1}\left({\mathrm{r⃗}}_{1}\right)=2\overset{\infty }{\underset{n=1}{\sum }}\lambda_{n}^{2}\;{\left[\varphi_{n}({\mathrm{r⃗}}_{1})\right]}^{2}$$ 41

and

$$\Phi \left(\mathrm{r⃗}\right)={\left(\overset{\infty }{\underset{n=1}{\sum }}\lambda_{n}\;{\left[\varphi_{n}(\mathrm{r⃗})\right]}^{2}\right)}^{2}$$ 42

giving rise to the reduced on-top two-electron density

$$\phi \left(\mathrm{r⃗}\right)={\left(\frac{\overset{\infty }{\underset{n=1}{\sum }}\lambda_{n}\;{\left[\varphi_{n}(\mathrm{r⃗})\right]}^{2}}{\overset{\infty }{\underset{n=1}{\sum }}\lambda_{n}^{2}\;{\left[\varphi_{n}(\mathrm{r⃗})\right]}^{2}}\right)}^{2}$$ 43

In practice, the infinite sums that enter eqs 32–34 are truncated at some n=N , producing (after the pertinent renormalization of the natural amplitudes) the approximate estimate

$${\mathrm{\phi ̃}}_{N}\equiv {\mathrm{\phi ̃}}_{N}\left(\mathrm{r⃗}\right)={\left(\frac{\overset{N}{\underset{n=1}{\sum }}\lambda_{n}\;{\left[\varphi_{n}(\mathrm{r⃗})\right]}^{2}}{\overset{N}{\underset{n=1}{\sum }}\lambda_{n}^{2}\;{\left[\varphi_{n}(\mathrm{r⃗})\right]}^{2}}\right)}^{2}\overset{N}{\underset{n=1}{\sum }}\lambda_{n}^{2}$$ 44

For N=1 , one recovers the HF result of *ϕ̃*_1_(*r⃗*) = 1, i.e. the previously mentioned fake ϕ­(*r⃗*) contrived from Ψ_*I*_.

In the case of the helium atom, λ_*n*_ < 0 for all *n* > 1 ^15,50^ so the truncation always results in an overestimation of ϕ. The extent of this overestimation is readily quantified with the help of the asymptotic evaluation of the addends that enter the three sums appearing in eq 35. As an example, consider the particular case of ϕ­(*r⃗*) at *r⃗* = 0, where only the *s*-type NOs contribute. These NOs and the corresponding NAs have the respective large-*k* asymptotics

45 $$\varphi_{k}^{s}\left(\mathrm{r⃗}\right)\approx {\mathrm{\varphi ̃}}_{k}^{s}\left(\mathrm{r⃗}\right)=\sqrt{\frac{1}{2\pi }}I_{1}^{-1/2}\;\frac{{\left[\Phi (r)\right]}^{1/16}}{r}\;\times \;\sin\left(k\;\pi \;I_{1}^{-1}\int_{0}^{r}{\left[\Phi (r^{\prime })\right]}^{1/8}\;dr^{\prime }\right)$$

and

46 $$\lambda_{k}^{s}\approx {\mathrm{\lambda ̃}}_{k}^{s}=-\frac{4I_{1}^{4}}{\pi^{3}}\;k^{-4}$$

where [note that ϕ­(*r*) ≡ ϕ­(*r⃗*) with *r* = |*r⃗*|]

47 $$I_{1}=\int_{0}^{\infty }{\left[\Phi (r)\right]}^{1/8}\;dr$$

Since it follows from eq 36 that

48 $$\varphi_{k}^{s}\left(0\right)\approx {\mathrm{\varphi ̃}}_{k}^{s}\left(0\right)=\sqrt{\frac{\pi }{2}}I_{1}^{-3/2}\;{\left[\Phi (0)\right]}^{3/16}\;k$$

the large- N asymptotics of ${\mathrm{\phi ̃}}_{N}\left(0\right)$ is determined by the addend $\lambda_{k}^{s}\;{\left[\varphi_{k}^{s}(0)\right]}^{2}∼k^{-2}$ that decays with *k* much slower than both ${\left(\lambda_{k}^{s}\right)}^{2}\;{\left[\varphi_{k}^{s}(0)\right]}^{2}∼k^{-6}$ and ${\left(\lambda_{k}^{s}\right)}^{2}∼k^{-8}$ . Accordingly, upon combining eqs 25, 32, 33, 35, 37, and 39, one obtains

$${\mathrm{\phi ̃}}_{N}\left(0\right)\approx {\mathrm{\phi ̃}}_{K(N)}^{s}\left(0\right)={\left(\frac{\overset{K\left(N\right)}{\underset{k=1}{\sum }}\lambda_{k}^{s}\;{\left[\varphi_{k}^{s}(\mathrm{r⃗})\right]}^{2}}{\overset{K\left(N\right)}{\underset{k=1}{\sum }}{\left(\lambda_{k}^{s}\right)}^{2}\;{\left[\varphi_{k}^{s}(\mathrm{r⃗})\right]}^{2}}\right)}^{2}\approx \phi \left(0\right)+\frac{16I_{1}\;{\left[\Phi (0)\right]}^{7/8}}{\pi^{2}\;{\left[\rho_{1}(0)\right]}^{2}}\;{K(N)}^{-1}$$ 49

where the number K(N) of the *s*-type NOs among the N NOs with the largest occupation numbers (equal to the squares of the respective NAs) is given by the asymptotic identity^15^

50 $$K\left(N\right)\approx {(\frac{\pi }{6I_{1}^{3}}\int {\left[\Phi (\mathrm{r⃗})\right]}^{3/8}d\mathrm{r⃗})}^{-1/3}N^{1/3}$$

The asymptotics [Disp-formula eq40] is confirmed by the numerical data derived from a highly accurate wave function of the ground state of the helium atom^50^ (Figure 2).

**Figure 2:**
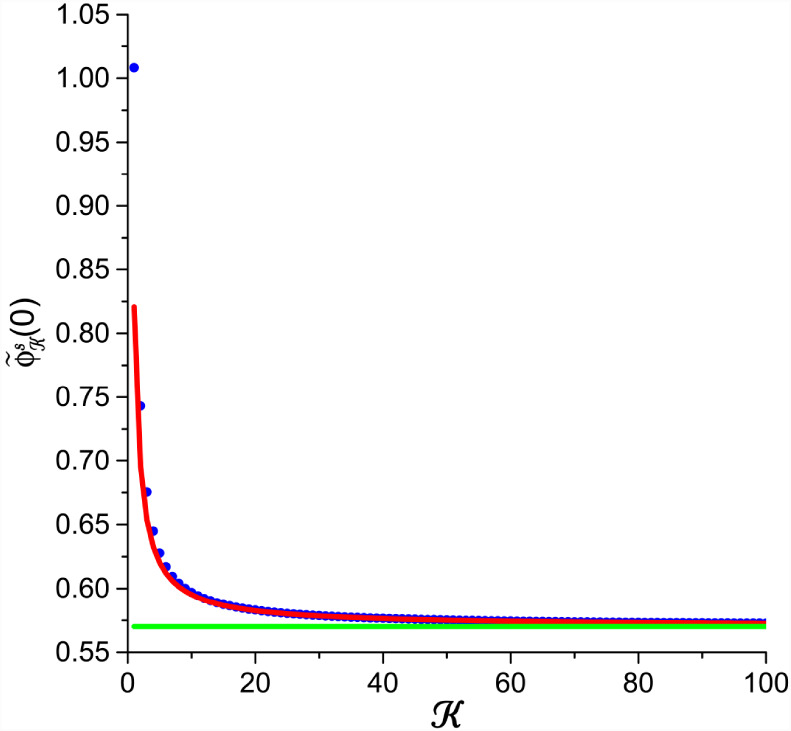
The approximate reduced on-top two-electron density ${\mathrm{\phi ̃}}_{K}^{s}\left(0\right)$ at the nucleus of the helium atom computed from K *s*-type natural orbitals with the largest occupation numbers (blue dots) and its large- K asymptotics (see eq 40) ${\mathrm{\phi ̃}}_{K}^{s}\left(0\right)\approx \phi \left(0\right)+0.250\;470\;K^{-1}$ (red line). The green line marks ϕ(0) ≈ 0.570 143.

Juxtaposing the $∼N^{-1/3}$ leading asymptotics of the truncation error in ϕ(0) that emerges from eqs 40 and 41 against the previously alluded to $∼N^{-1}$ power law governing the accuracy of the electronic energy^18^ reveals the extremely slow convergence of the on-top two-electron density and its reduced variant to the CBS limit. This $N^{-1/3}$ scaling of the error is expected to carry over to the values of ϕ(0) obtained from actual electronic structure calculations, though with different multiplicative factors due to the counts of the basis functions with different angular momenta not necessarily following those of the NOs ordered according to their occupation numbers.

As the first weakly occupied NO of the ground state of the He atom is of the *s* type,^50^ FCI calculations employing even small basis sets furnish approximate values of ϕ(0) that do not exceed one. Thanks to this fortuitous combination of the NO ordering and the nature of the dependence of ${\mathrm{\phi ̃}}_{K}^{s}\left(0\right)$ on K shown in Figure 2, the reduced on-top two-electron density computed within, e.g., the CASSCF­(2,2) method^42^ turns out to be roughly similar to its exact counterpart displayed in Figure 1. An entirely different situation is encountered in the case of the ground state of the H_2_ molecule, in which only the σ_*g*_-type NOs contribute to the reduced on-top two-electron density $\phi \left(0\right)\equiv \phi \left(\mathrm{r⃗}\right){|}_{\mathrm{r⃗}=(0,0,0)}$ at the bond midpoint. Therefore, as the first weakly occupied NO of this system is of the σ_*u*_ type,^15^ it follows from eq 35 that the CASSCF­(2,2) approach inevitably produces 1 < ϕ(0) < 2 irrespective of the nuclei-centered one-electron basis set used.^41,43^ This approximate ϕ(0) increases monotonically with the internuclear distance *R*_*HH*_ as the occupation number of the σ_*g*_-type NOs tends to $\frac{1}{2}$ at the bond dissociation limit.^41,43^

These values are both spurious and unphysical, as the true reduced on-top two-electron densities $\phi \left(z\right)\equiv \phi \left(\mathrm{r⃗}\right){|}_{\mathrm{r⃗}=(0,0,z)}$ along the bond axis do not resemble at all their approximate counterparts obtained with oversimplistic approaches such as CASSCF­(2,2). Thus, the true ϕ­(*z*) pertaining to the equilibrium distance *R*_*HH*_ = 1.4011 peaks at ca. 0.495 (Figure 3a), which is less than the *minimum* value of ca. 0.503 assumed by the approximate ϕ­(*z*) produced by a model that involves the CASSCF(2,2) formalism applied to linear combinations of two *s*-type hydrogenic orbitals with optimized exponents.^41^ Not surprisingly, the latter ϕ­(*z*) exceeds one at *z* = 0 and so does its incarnation employing a more sophisticated basis set.^43^ As predicted, the convergence of the approximate ϕ­(*z*) computed within the FCI formalism to the CBS limit is exceedingly slow, the ϕ(0) values obtained with the cc-pVTZ and cc-pV5Z basis sets equaling ca. 0.681 and 0.586, respectively.

**Figure 3:**
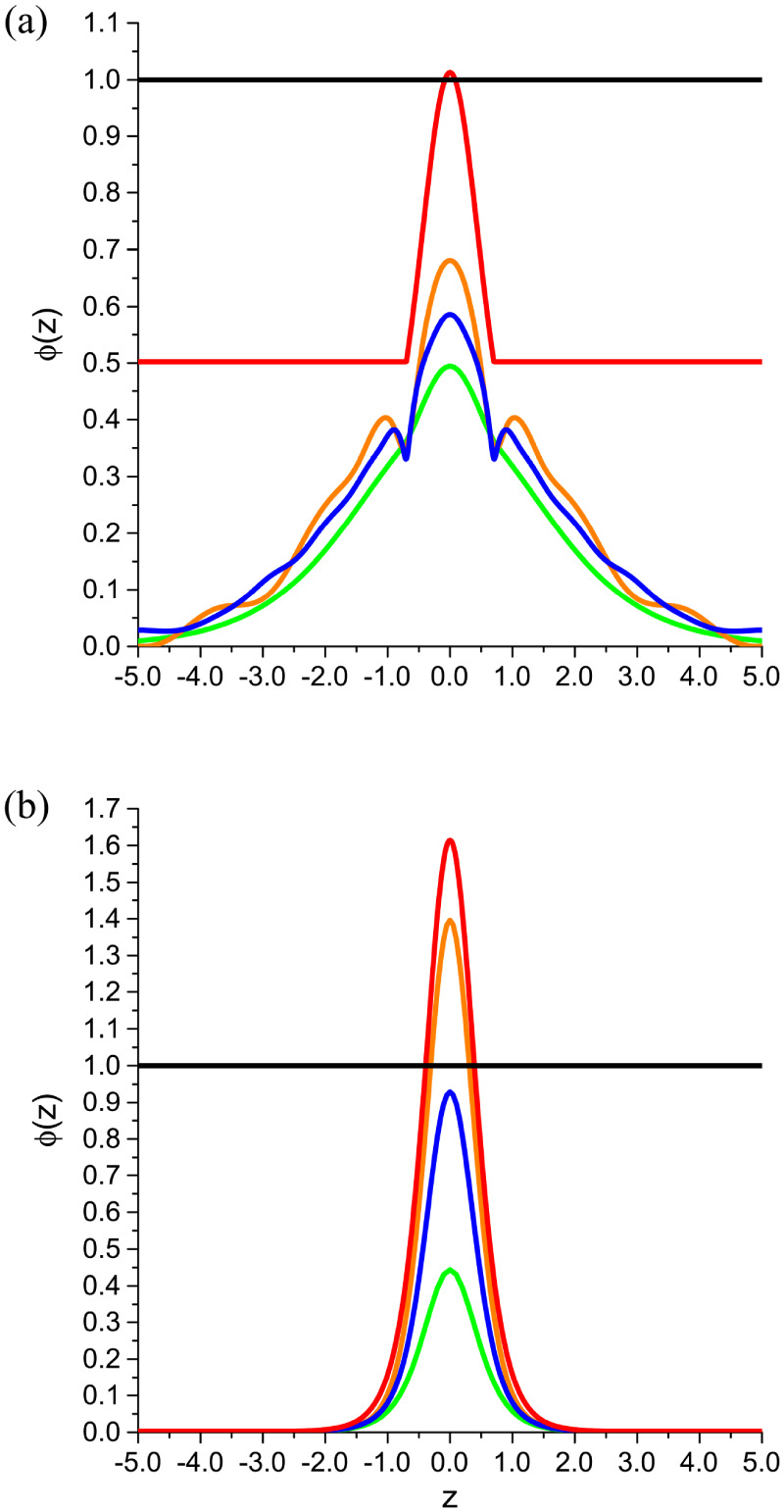
The reduced on-top two-electron density ϕ(z)≡ $\phi \left(\mathrm{r⃗}\right){|}_{\mathrm{r⃗}=(0,0,z)}$ of the ground state of the H_2_ molecule with the internuclear distances: a) *R_HH_* = 1.4011 and b) *R_HH_* = 5.0. The essentially exact ϕ­(*z*) (green), the ϕ­(*z*) obtained with the minimal-basis-set model (red), and the ϕ­(*z*) contrived from Ψ*_I_* (black) are displayed together with those computed at the FCI/cc-pVTZ (orange), and FCI/cc-pV5Z (blue) levels of theory. The hydrogen nuclei are positioned at $z=\left(0,0,\pm \frac{1}{2}R_{HH}\right)$ .

Another set of differences is encountered at *R*_*HH*_ = 5.0 (Figure 3b). Although the oscillatory artifacts present in the ϕ­(*z*) computed at the FCI/cc-pVTZ and FCI/cc-pV5Z levels of theory are no longer evident, its convergence to the CBS limit is noticeably slower, with both the minimal-basis-set model and the FCI/cc-pVTZ level of theory producing the values of ϕ(0) greater than one (ca. 1.615 and 1.396, respectively), and the FCI formalism used in conjunction with the cc-pV5Z still overestimating it by a factor of ca. 2.094 (ca. 0.929 vs 0.444). These observations are in line with the data previously published for *R*_*HH*_ = 4.0.^43^

The true ϕ(0) evolves with *R*_*HH*_ in a manner strikingly different from that of its approximate minimal-basis-set counterpart (Figure 4). Whereas, as explained above, the latter increases monotonically with *R*_*HH*_ and assumes values greater than one, the former never exceeds its united-atom limit of ca. 0.570 143. This discrepancy is not surprising in light of the failure of the minimal-basis-set model to reproduce, even qualitatively, the detailed picture of electron correlation in the ground state of the H_2_ molecule that is provided by the natural densitals.^6^

**Figure 4:**
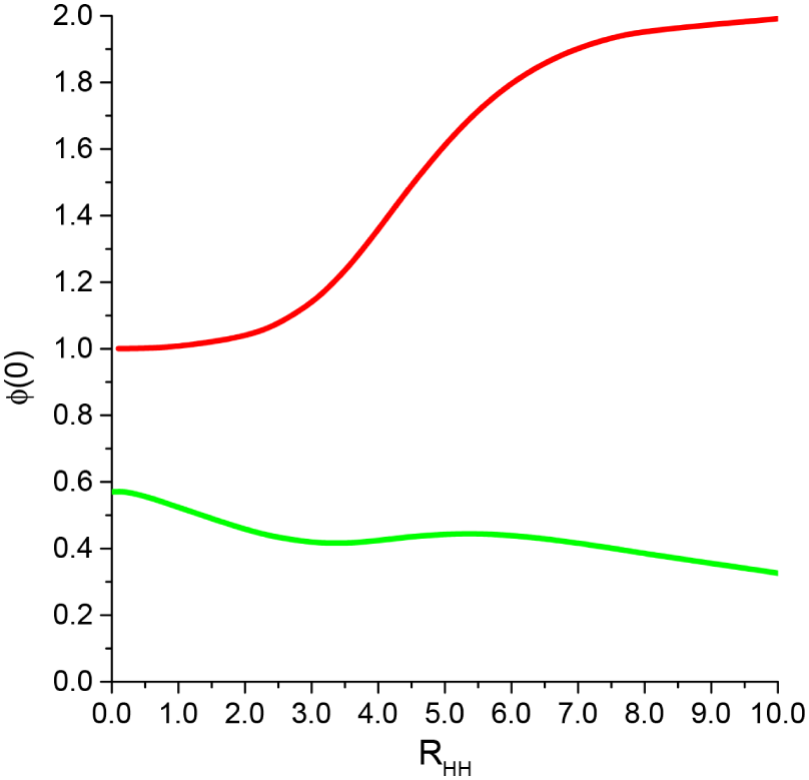
The dependence of the reduced on-top two-electron density $\phi \left(0\right)\equiv \phi \left(\mathrm{r⃗}\right){|}_{\mathrm{r⃗}=(0,0,0)}$ pertaining to the ground state of the H_2_ molecule on the internuclear distance *R_HH_*. The essentially exact ϕ(0) (green) and that obtained with the minimal-basis-set model (red) are displayed.

Three salient properties of the true reduced on-top two-electron density pertaining to the ground state of the H_2_ molecule emerge from the above observations: 1) its maximum, attained at the bond midpoint, is as blind to the nondynamical correlation as it is exquisitely sensitive to the dynamical one; 2) it does not partition the Cartesian space into domains delineated by the boundary condition ϕ­(*r⃗*) = 1; and 3) it does not allow discerning different bonding scenarios from its variation along the bond axis [described by the essentially featureless Gaussian-like function ϕ­(*z*)]. These properties of ϕ­(*r⃗*) invalidate several published assertions and concepts formulated on the basis of unwarranted generalizations stemming from the analysis of the data produced by grossly inadequate treatments of electron correlation. In particular, the claims that “at the bond midpoint [...] correlation increases the probability of finding the electrons in the same place relative to an uncorrelated wave function” and that the behavior of ϕ(0) “may be a way to quantify bond breaking in molecules”^41^ are patently wrong. The idea of the spatial domains with ϕ­(*r⃗*) < 1 and ϕ­(*r⃗*) > 1 being the loci of, respectively, “the suppression of dynamic correlation (SDC) with nondynamic correlation” and “the enhanced dynamic correlation (EDC) of the electrons”,^43^ does not fare any better. Taken literally, the assertions that “in spatial regions where *ϕ*(*r⃗*) falls below 1, the nondynamic correlation suppresses the dynamic correlation” and “in spatial regions where *ϕ*(*r⃗*) > 1, [...] the dynamic correlation is enhanced”^44^ imply the suppression of dynamical correlation throughout the entire Cartesian space and at any internuclear distance, flying in the face of the profound reduction of ϕ­(*r⃗*) upon proper inclusion of dynamical correlation and the marginal influence of variations in the nondynamical correlation on ϕ(0⃗). To reiterate, although there is no doubt that the electron correlation in the ground state of the H_2_ molecule has both the dynamical and nondynamical components (in proportions that vary with the internuclear distance), the former overwhelmingly controls the magnitude of ϕ­(*r⃗*), plain and simple.

Strictly speaking, the above conclusions, which also concern the concept of the so-called correlons,^46,47^ pertain only to the ground state of the H_2_ molecule. However, taking into account the major changes in ϕ­(*r⃗*) of this system brought about by correct treatment of the short-range correlation effects, analogous qualitative transmutations of the reduced on-top two-electron densities derived from insufficiently accurate wave functions of other species^42–47^ are expected to be a rule rather than an exception. In particular, with the Coulombic repulsion being a powerful suppressor of the electron-electron coalescence, the true ϕ­(*r⃗*) of the ground states are almost certainly devoid of domains with ϕ­(*r⃗*) > 1, the same morphology of ϕ­(*r⃗*) being quite likely for the excited states. Confirmation of these expectations is contingent upon the availability of highly accurate electronic wave functions of several atoms and molecules.

In summary, the reduced on-top two-electron densities of the ground states of the H_2_ molecule with arbitrary internuclear separations (and most probably also those of general systems) have properties crucial to their proper interpretation that are exceedingly difficult to reproduce at the levels of theory known to suffice for accurate predictions of, e.g., the electronic energies. These densities are poor in features of relevance to the analysis of electronic wave functions, making them ill-suited for employment as an interpretive tool. Even worse, in a textbook example of availability bias, the spurious features of their inaccurate counterparts have prompted unsubstantiated and misleading descriptions of electron correlation and chemical bonding. With those features being nothing but red herrings, the efforts to compute inaccurate on-top two-electron densities^53^ are merely exercises in numerical calculations of doubtful practical significance.

## 3 Can the Accuracy of the On-Top Two-Electron Density Be Improved at Little Computational Cost?

The slow convergence of the on-top two-electron density and its reduced variant to the CBS limit translates into the high cost of its reliable computation, prompting the question of whether it can be alleviated with the convergence acceleration techniques commonly applied to other electronic properties. In general, these techniques fall into two broad categories, namely those based upon extrapolations and those specifically designed for the expectation values of operators involving the three-dimensional Dirac delta. By virtue of eq 20, the performance assessment of these techniques is conveniently carried out in the case of Φ by quantifying its global accuracy with the error in the respective intracule density I̅(0) at the electron-electron coalescence point. Accordingly, the approximate values of I̅(0) have been computed within the FCI formalism used in conjunction with several basis sets and compared with their essentially exact counterparts furnished by the previously published benchmark-quality electronic structure calculations.^50,51^ These values, which are listed in Table 2, have, in turn, been input into the working equations of various convergence acceleration schemes.

**Table 2:** The Exact and Approximate Ground-State Intracule Densities I̅(0) at the Electron-Electron Coalescence Point^a^

		Basis set^b^				
Species^c^	Exact^d^	cc-pVDZ	cc-pVTZ	cc-pVQZ	cc-pV5Z	cc-pV6Z
He	0.106345	0.147446 [39]	0.132015 [24]	0.124646 [17]	0.120563 [13]	0.118001 [11]
Li	0.544337	0.783985 [44]	0.727742 [34]	0.721938 [33]	0.697474 [28]	
Be	1.605697	2.089594 [30]	2.061493 [28]	1.957809 [22]	1.919724 [20]	
Li^–^	0.544409	0.790639 [45]	0.728013 [34]	0.722196 [33]	0.697617 [28]	
H_2_	0.016720	0.025453 [52]	0.022524 [35]	0.020945 [25]	0.019985 [20]	0.019401 [16]
$H_{3}^{+}$	0.018335	0.027769 [51]	0.024788 [35]	0.022992 [25]	0.021885 [19]	0.021263 [16]
LiH	0.549304	0.792646 [44]	0.732782 [33]	0.724353 [32]		

a The approximate values of I̅(0) computed within the FCI formalism are followed by their relative percentage errors in square brackets.

b The basis sets used are those listed in the Basis Set Exchange.^54^

c The internuclear distances read: *R*_*HH*_ = 1.4011 (H_2_), *R*_*HH*_ = 1.65 ( $H_{3}^{+}$ with an equilateral triangle geometry), and *R*_*LiH*_ = 3.015.

d The essentially exact values derived directly from benchmark-quality wave functions.^50,51^

The $∼N^{-1/3}$ leading asymptotics of the truncation error, rigorously proven [see eqs 40 and 41] for the reduced on-top two-electron density ϕ(0) at the nucleus of the helium atom in its ground state, can also be readily demonstrated for I̅(0) derived from the wave function [Disp-formula eq31]. Combining eqs 20 and 33 produces

$${\mathrm{I̅}}_{N}\left(0\right)=\int \;{\left(\overset{N}{\underset{n=1}{\sum }}\lambda_{n}\;{\left[\varphi_{n}\left(\mathrm{r⃗}\right)\right]}^{2}\right)}^{2}d\mathrm{r⃗}=\int \;[\;{\left(\overset{\infty }{\underset{n=1}{\sum }}\lambda_{n}\;{\left[\varphi_{n}\left(\mathrm{r⃗}\right)\right]}^{2}\right)}^{2}-2\;\left(\overset{\infty }{\underset{n=1}{\sum }}\lambda_{n}\;{\left[\varphi_{n}\left(\mathrm{r⃗}\right)\right]}^{2}\right)\;\left(\overset{\infty }{\underset{n=N+1}{\sum }}\lambda_{n}\;{\left[\varphi_{n}\left(\mathrm{r⃗}\right)\right]}^{2}\right)+\;{\left(\overset{\infty }{\underset{n=N+1}{\sum }}\lambda_{n}\;{\left[\varphi_{n}\left(\mathrm{r⃗}\right)\right]}^{2}\right)}^{2}]\;d\mathrm{r⃗}\approx \;\mathrm{I̅}\left(0\right)-2\overset{\infty }{\underset{n=N+1}{\sum }}\lambda_{n}\int \;{\left[\varphi_{n}\left(\mathrm{r⃗}\right)\right]}^{2}\;{\left[\Phi \left(\mathrm{r⃗}\right)\right]}^{1/2}\;d\mathrm{r⃗}$$ 51

for the approximate ${\mathrm{I̅}}_{N}\left(0\right)$ arising from N NOs with the largest occupation numbers. Since, for sufficiently large *n*,^15^

52 $$\lambda_{n}\approx {\mathrm{\lambda ̃}}_{n}=-{(\frac{2^{1/2}}{3\pi^{5/4}}\int {[\Phi (\mathrm{r⃗})]}^{3/8}\;d\mathrm{r⃗})}^{4/3}n^{-4/3}$$

and (in the case of systems with spherical symmetry)

53 $$\int {[\varphi_{n}(\mathrm{r⃗})]}^{2}\;{[\Phi (\mathrm{r⃗})]}^{1/2}\;d\mathrm{r⃗}\approx \frac{1}{I_{1}}\int_{0}^{\infty }{[\Phi (r)]}^{5/8}\;dr$$

the asymptotics

$${\mathrm{I̅}}_{N}\left(0\right)\approx \mathrm{I̅}\left(0\right)+\frac{2^{5/3}}{3^{1/3}\pi^{5/3}}\;I_{1}^{-1}\;{\left(\int {[\Phi (\mathrm{r⃗})]}^{3/8}\;d\mathrm{r⃗}\right)}^{4/3}\times \;(\int_{0}^{\infty }{[\Phi (r)]}^{5/8}\;dr)\;N^{-1/3}$$ 54

holds (at least for the ^1^S states of the helium isoelectronic series).

In light of this asymptotics, an extrapolation scheme based upon the approximate formula

55 $${\mathrm{I̅}}_{K}^{•}\left(0\right)\approx \mathrm{I̅}\left(0\right)+\frac{A}{K+B}$$

where ${\mathrm{I̅}}_{K}^{•}\left(0\right)$ is the approximate I̅(0) computed with the one-electron basis functions comprising the K th member of a systematically constructed family of basis sets (for which one assumes constant values of *A* and *B*), appears to be worthy of consideration (note , however, the tenuous connection between the truncation errors for the ^1^S states and the errors pertaining to sets of nuclei-centered basis functions in general systems^17^). Combining this equation for three consecutive K s yields the desired extrapolation scheme

56 $$\mathrm{I̅}\left(0\right)\approx \frac{\left[{\mathrm{I̅}}_{K+1}^{•}\left(0\right)-{\mathrm{I̅}}_{K}^{•}\left(0\right)\right]\;{\mathrm{I̅}}_{K+2}^{•}\left(0\right)-\left[{\mathrm{I̅}}_{K+2}^{•}\left(0\right)-{\mathrm{I̅}}_{K+1}^{•}\left(0\right)\right]\;{\mathrm{I̅}}_{K}^{•}\left(0\right)}{\left[{\mathrm{I̅}}_{K+1}^{•}\left(0\right)-{\mathrm{I̅}}_{K}^{•}\left(0\right)\right]-\left[{\mathrm{I̅}}_{K+2}^{•}\left(0\right)-{\mathrm{I̅}}_{K+1}^{•}\left(0\right)\right]}$$

Inspection of the extrapolated values of I̅(0) presented in Table 3 reveals a mixed performance of this scheme. Whereas substantial gains in accuracy are realized for two-electron systems, no overall improvement is observed in the case of the three- and four-electron species. Therefore, it appears that the intracule electron densities, and thus their (reduced) on-top two-electron counterparts, obtained from families of standard basis sets are, in general, not amenable to error reduction via extrapolation to the CBS limit. Most likely stemming from the paucity of basis functions per each angular momentum (which precludes a faithful reproduction of the NOs), this disappointing performance is not a failure of the extrapolation scheme *per se*. Indeed, significantly more accurate values of both the raw and extrapolated I̅(0) are obtained with the previously reported even-tempered basis sets comprising uncontracted *s*-, *p*-, and *d*-type Gaussian primitives.^55^ In particular, for the helium atom, the (20s), (20s10p), and (20s10p10d) basis sets of this type yield I̅(0) equaling, respectively, ca. 0.155 793, 0.128 656, and 0.121 271, the second and the third of these values being more accurate than, respectively, those produced by the cc-pVTZ and cc-pVQZ sets (the former including the *d*-type one-electron basis functions and the latter the *f*-type ones). When input into eq 47, these values yield the CBS-limit estimate of ca. 0.108 365, whose relative percentage error of ca. 1.9% is slightly smaller than that produced by the extrapolation involving the cc-pVDZ, cc-pVTZ, and cc-pVQZ basis sets (Table 3). In the case of the beryllium atom, the raw values of the approximate I̅(0) read ca. 1.941 307, 1.764 696, and 1.713 850, the first of them being already better than that furnished by the cc-pVQZ basis set (which includes the *g*-type functions). From these raw values, one obtains the CBS-limit estimate of ca. 1.621 893, which, in terms of the relative percentage error (ca. 1.0%), is more accurate than that computed for the helium atom.

**Table 3:** The Ground-State Intracule Densities I̅(0) at the Electron-Electron Coalescence Point Extrapolated to the CBS Limit^a^

	The range of basis sets used in extrapolation		
Species	cc-pVDZ–cc-pVQZ	cc-pVTZ–cc-pV5Z	cc-pVQZ–cc-pV6Z
He	0.103809 [−2.4]	0.106332 [0.0]	0.106805 [0.4]
Li	0.714797 [31.3]	0.737158 [35.4]	
Be	2.138589 [33.2]	1.837415 [14.4]	
Li^–^	0.715187 [31.4]	0.737437 [35.5]	
H_2_	0.015681 [−6.2]	0.016034 [−4.1]	0.017014 [1.8]
$H_{3}^{+}$	0.015742 [−14.1]	0.017232 [−6.0]	0.019045 [3.9]
LiH	0.713163 [29.8]		

a See the footnotes to Table 2.

Being the expectation values of operators comprising either single three-dimensional Dirac deltas (in the cases of ρ_1_, I , and I̅ ) or their products (in the cases of ρ_2_ and Φ), the various incarnations of the one- and two-electron densities can, in principle, be obtained from alternative expressions that, while being fully equivalent for the eigenfunctions of the electronic Hamiltonian, are far less sensitive to the errors inherent in approximate wave functions.^56^ Unfortunately, the utility of both the Hiller-Sucher-Feinberg^57^ and Drachman^58^ identities that underlie these alternate expressions is somewhat limited, the former lacking an analogue applicable to products of Dirac deltas (and thus not being appropriate for the computation of either ρ_2_ or Φ) and the latter giving rise to a formula for Φ whose excessive complexity precludes efficient numerical evaluation. Consequently, these convergence acceleration formalisms are of no relevance to the accuracy improvement of the on-top two-electron density.

Taking advantage of the known cusp conditions at coalescence points,^59^ the “plain capping” formalism reduces the errors in approximate $\rho_{1}\left({\mathrm{r⃗}}_{1}\right)$ , I̅(r) , and Φ­(*r⃗*) at, respectively, *r⃗*_1_ coinciding with the position of a nucleus, *r* = 0, and an arbitrary *r⃗* outside of small regions about nuclei.^51^ In the case of the spherically-averaged intracule density, the original equations of this formalism can be reformulated to yield a compact expression

57 $${\mathrm{I̅}}^{cap}\left(0\right)=\underset{r}{\min}\mathrm{I̅}\left(r\right)\;\exp\left(-\frac{r}{2}\left[\frac{d\ln\mathrm{I̅}\left(r\right)}{dr}+1\right]\right)$$

where the minimum corresponding to the smallest positive value of *r* is taken. For approximate intracule densities at the electron-electron coalescence point that are reasonably close to their exact counterparts, replacing I̅(0) with ${\mathrm{I̅}}^{cap}\left(0\right)$ results in the reduction of error by between one to two orders of magnitude.^51^ Unfortunately, in all of the cases listed in Table 2 (as well as in those involving the aforementioned even-tempered basis sets), the function that enters the r.h.s. of eq 48 turns out to have no minimum at finite positive-valued *r*, indicating that the basis sets feasible within the limitations imposed by the computational cost of the FCI formalism produce spherically-averaged intracule densities whose flaws in the vicinity of the electron-electron coalescence point are too severe to be rectifiable by plain capping. This conclusion is borne out by the properties of the approximate I̅(r) computed with these basis sets. Whereas the cusp condition [Disp-formula eq19] imposes the presence of both a minimum at *r* = 0 and a maximum at some *r* > 0, the majority of the values of I̅(0) compiled in Table 2 pertain to maxima (see, e.g., Figure 5a), the only exceptions being the spherically-averaged intracule densities of the H_2_ and $H_{3}^{+}$ species obtained with the cc-pVTZ, cc-pVQZ, cc-pV5Z, and cc-pV6Z basis sets. Moreover, among the aforementioned even-tempered basis sets, only that including the *d*-type function yields I̅(r) with a minimum at *r* = 0 and only for the helium atom. Even in those instances, the minima at *r* = 0 are apparently too shallow (see, e.g., Figure 5b) for the “plain capping” formalism to offer any improvement.

**Figure 5:**
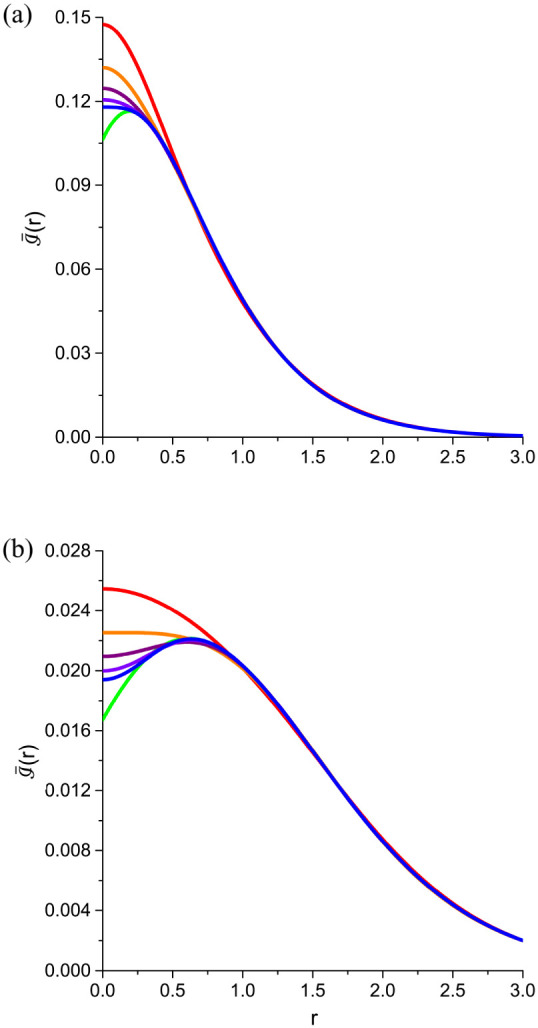
The spherically-averaged intracule density I̅(r) of the ground state of a) the helium atom and b) the H_2_ molecule with *R_HH_* = 1.4011. The essentially exact I̅(r) (green) is displayed together with those computed at the FCI/cc-pVDZ (red), FCI/cc-pVTZ (orange), FCI/cc-pVQZ (purple), FCI/cc-pV5Z (violet), and FCI/cc-pV6Z (blue) levels of theory.

In summary, the on-top two-electron densities calculated within the FCI formalism used in conjunction with computationally tractable basis sets are refractory to accuracy improvement. In particular, their errors cannot be reduced by the extrapolation to the CBS limit unless the underlying family of basis sets comprises large numbers of uncontracted primitives. The techniques of Drachmanization and HSFization, which afford impressive reduction of errors in the one-electron and intracule densities, are not applicable to Φ, whereas the “plain capping” approach fails, apparently due to excessive deviations of the raw I̅(r) from their exact counterparts. It should also be emphasized that the global errors of Φ, quantified by those of I̅(0) , roughly reflect the local ones, an example at hand being the Φ of the ground state of the H_2_ molecule at *R*_*HH*_ = 1.4011 computed at the FCI/cc-pV5Z level of theory, which has the global relative percentage error of ca. 20% (Table 2) and that at the bond midpoint amounting to ca. 18% (Figure 3a).

## 4 Summary of Findings and Concluding Remarks

In the past decade or two, the on-top two-electron density Φ and its reduced variant ϕ have become ubiquitous in electronic structure theory. Controlling the properties of natural orbitals and their occupation numbers within the weak-occupation regime,^15^ Φ enters the expressions for the asymptotic estimates of errors in electronic properties due to the basis-set truncation,^17,18^ facilitating the construction of approximate CBS-limit extrapolation schemes. By the same token, it governs the symmetry incidences of natural orbitals.^19^ Within the conventional Kohn–Sham formalism, Φ provides a mechanism for escaping the symmetry dilemma,^26^ whereas within its multiconfigurational generalization, ϕ serves as a conduit for the inclusion of nondynamical correlation effects.^30,38^ In addition, Φ appears in the expression for the relativistic correction to the nonrelativistic electronic energy^60,61^ (whose convergence to the respective CBS limits has been repeatedly studied in the case of explicitly correlated basis sets^51,62–64^ ) whereas in the case of the homogeneous electron gas, ϕ is proportional to the position-independent pair-correlation function at the vanishing interelectron distance.^22^ In all of those instances, Φ and ϕ have opened new vistas for understanding and prediction of the electronic properties of atoms, molecules, and extended systems. On the other hand, in a development detrimental to the widely perceived reliability of quantum-chemical calculations, spurious features of grossly inaccurate approximations to Φ and ϕ have spurred formulations of invalid interpretive concepts that have led to misleading inferences about chemical bonding and electron correlation.^41–47^

Like its parent (i.e., ρ_2_) and companion (i.e., $D_{2}$ , I , and I̅ ) quantities, Φ is typically computed in a conventional manner from the two-electron reduced density matrix ^2^Γ expressed in terms of a finite number of one-electron basis functions. The incompleteness of this basis set imparts the truncation error into the approximate Φ that, as unequivocally demonstrated by the research reported in this paper, decays exceedingly slowly with the number N of the basis functions included in the construction of ^2^Γ. In the instance of these basis functions being natural orbitals, rigorous asymptotic analysis provides convincing arguments for the large- N behavior of this decay being $∼N^{-1/3}$ . As this decay is much slower than that of $∼N^{-1}$ pertaining to the truncation error of the electronic energy, the basis sets commonly employed in reliable predictions of other electronic properties are utterly inadequate in the case of Φ. Even worse, the results of test calculations involving the full configuration interaction formalism (which is exact within a given basis set) underline the importance of saturating the subsets of basis functions with individual angular momenta *l* in high-*l* nuclei-centered basis sets. Most of the convergence acceleration techniques commonly applied to other electronic properties do not work for Φ, the only exception being a simple extrapolation to the CBS limit that, however, is predicated upon the aforementioned basis-subset saturation.

In summary, the on-top two-electron densities that stem from basis sets with sizes limited by computational cost considerations inherently suffer from gross inaccuracies. Ranging from none to crucial, the consequences of these inaccuracies depend strongly on the context in which those densities are employed. Thus, the accuracy of the Φ furnished by the modified KS formalism designed to escape the symmetry dilemma, which is locally low (see Figure 1 for an example) but surprisingly high globally [as evidenced by the relatively small errors of various related integrals such as I̅(0) ] has no obvious influence on that of the concomitantly computed one-electron density and electronic energy. The accuracy of the Φ that enters the multiplicative constants in the asymptotics of the truncation errors of various electronic properties is of minor importance since, as already mentioned, these constants are subject to empirical adjustments in the resultant extrapolation schemes. As revealed by the present research, a paradoxical situation is encountered in the case of the MC-PDFT multiconfigurational generalization of the KS formalism, in which the poor accuracy of the Φ affected by the inclusion of only a small fraction of the dynamical correlation actually works to the advantage of that approach, tentatively rescuing it from an occult double-counting problem (though certainly not strengthening its weak theoretical foundations). In the context of the relativistic correction to the nonrelativistic electronic energy, the accuracy of Φ is of crucial importance for obtaining reliable predictions of properties that are juxtaposed with those measured in extremely precise experiments. Similarly, the availability of highly accurate Φ facilitates consistency checks of both analytic and numerical approaches to electron correlation in the homogeneous electron gas.^65,66^

The diverse facets of the on-top two-electron density are readily reconciled insofar as one maintains the distinction between the true Φ, which is obtainable only from high-quality electronic structure calculations, and that appearing in various incarnations of the KS formalism. Whereas the former is an observable of physical relevance, the latter is merely a method-specific auxiliary to which no physical meaning should be attached. Failure to recognize this distinction opens an avenue to the employment of physically irrelevant quantities as interpretive tools. As the properties of such quantities are exceedingly sensitive to the methods (such as the combination of the basis set and the density functional) involved in their calculation, they can be easily manipulated, thus obfuscating rather than elucidating the details of quantum-chemical phenomena. This assertion is supported by the aforediscussed inefficacy of the concepts based upon grossly inaccurate Φ applied to even the simplest systems, such as the H_2_ molecule.

The options for a reliable numerical evaluation of the on-top two-electron density are very limited at present. The high computational cost of the FCI formalism imposes severe constraints on the sizes of viable one-electron basis sets, ruling out acceptable accuracy of the resulting Φ. Although one is tempted to consider less demanding approaches (such as the coupled cluster theory) that would permit the application of much larger basis sets, the requirement of additive separability (a.k.a. size consistency) of the pertinent one- and two-electron properties mandates the construction of ^2^Γ within the energy-derivative formalism that is known to yield incorrectly normalized two-electron density.^55^ Thus, it appears that the employment of explicitly correlated basis functions is a prerequisite for avoiding unacceptably large errors in the computed Φ. However, it has to be emphasized that Φ is a feature-poor quantity that carries much less detailed information than, e.g., the natural densitals and their amplitudes [the signs of the latter governing the morphology of Φ­(*r⃗*) via eq 26]. Since its computation inevitably proceeds via the expensive construction of ^2^Γ from which the natural densitals are readily obtained, there is little reason for using Φ as an interpretive tool.

This observation concludes the cautionary tale of two-electron densities computed with one-electron basis sets and of their employment in quantum-chemical formalisms. If there is a lesson that emerges from this tale, it reads: when constructing interpretive tools for the analysis of electronic structures, never build them around quantities that are notoriously difficult to calculate and whose accuracy is refractory to improvement.

## APPENDIX

### Computational Details

The essentially exact numerical values of various quantities reported in this paper have been derived from the previously published benchmark-quality wavefunctions.^6,50,51^ Their approximate counterparts pertaining to one-electron basis sets used in conjunction with the FCI formalism have been computed with a modified version of the Knowles–Handy code^67,68^ reading the data generated by the Gaussian 09 suite of programs^69^ and the in-house DMn code.^70^ With the exception of the even-tempered ones, the basis sets have been obtained from the Basis Set Exchange.^54^ The spherically-averaged intracule densities have been computed with an algorithm involving special exact quadratures.^71^
